# Identification and functional study of GATA4 gene regulatory variants in type 2 diabetes mellitus

**DOI:** 10.1186/s12902-021-00739-0

**Published:** 2021-04-17

**Authors:** Liangcai Ding, Mengdi Cai, Lu Chen, Han Yan, Shicheng Lu, Shuchao Pang, Bo Yan

**Affiliations:** 1 Center for Molecular Medicine, Yanzhou People’s Hospital, Jining Medical University, Jining, 272100 Shandong China; 2 Division of Endocrinology, Yanzhou People’s Hospital, Jining Medical University, Jining, 272100 Shandong China; 3 Shandong Provincial Sino-US Cooperation Research Center for Translational Medicine, Affiliated Hospital of Jining Medical University, Jining Medical University, 89 Guhuai Road, Rencheng District, Jining City, 272029 Shandong China

**Keywords:** Type 2 diabetes mellitus, Genetics, GATA4, Regulatory variants

## Abstract

**Background:**

Type 2 diabetes mellitus (T2D) is a common and complex disease. Dysfunction of pancreatic β cells, which cannot release sufficient insulin, plays a central role in T2D. Genetics plays a critical role in T2D etiology. Transcription factor GATA4 is required for the pancreatic development, and GATA4 gene mutations are implicated in neonatal or childhood-onset diabetes. In this study, we aimed to investigate whether regulatory variants in GATA4 gene may change GATA4 levels, conferring susceptibility to T2D development.

**Methods:**

The promoter region of GATA4 gene was analyzed by targeted sequencing in T2D patients (*n* = 255) and ethnic-matched controls (*n* = 371). Dual luciferase activity assay was used for functional study, and EMSA (electrophoretic mobility shift assay) was performed for detecting transcription factor binding.

**Results:**

Thirteen regulatory variants including 5 SNPs were identified. A novel heterozygous variant (32124C > T) and one SNP [31487C > G (rs1053351749)] were only identified in T2D. Both regulatory variants significantly affected GATA4 gene promoter activity in cultured HEK-293 and INS-1 cells. Furthermore, the variant (32124C > T) evidently enhanced the binding of unknown transcriptional activator.

**Conclusions:**

Our data suggested that GATA4 gene regulatory variants may contribute to T2D development as a rare risk factor.

## Background

Type 2 diabetes mellitus (T2D) is a common and complex disease and caused by interactions between genetic and environmental factors [1]. Type 2 DM is mainly caused by inflammation due to overloading of adipous tissue. The resulting insulin resistance causes overstimulation of pancreatic beta-cells for compensation. This chronic overproduction, islet hyperplasia and inflammation of pancreatic islets eventually lead to beta-cell dysfunction and overt T2D. To date, genetic studies have associated hundreds of genetic loci with T2D susceptibility. A highly polygenic architecture of T2D has been established, which is dominated by common alleles with small and cumulative effects [2]. Since the identified genetic loci collectively accounts for a small portion of T2D cases, genetic etiology for T2D needs to be further investigated. Low-frequency and rare sequence variants have been implicated in T2D risk by modulating β-cell mass and function [3]. Therefore, genetic studies indicate that T2D is a highly heterogeneous and polygenic disease [4].

Transcription factor GATA4 plays essential roles in many cellular processes [5–8]. GATA4 gene is expressed in mesoderm and endoderm derived tissues, including pancreas in mice [5]. In human, GATA4 gene expressed is detected in heart, liver, pancreas, stomach, small intestines, gall bladder, ovary and testis [6]. Mice with GATA4 gene deletion die before birth, mainly due to severe defects in heart morphogenesis and ventral foregut closure [7, 8]. In mouse embryos, GATA4 regulates development of pancreatic progenitors, and morphogenesis of pancreas [9, 10]. Pancreatic-specific deletion of GATA4 gene results in mild pancreatic defects. Double deletion of GATA4 and GATA6 in pancreas causes severe agenesis [10]. In mice, GATA4 deficiency causes ectopic pancreas, which contains all three pancreatic lineage cells [11]. Therefore, GATA4 is required for pancreatic development.

Accumulating human studies indicate that defective pancreatic β-cells plays a central role in T2D pathogenesis [12]. A significant proportion of genetic variants for T2D risk impacts pancreatic islet cell function and insulin secretion [13]. Mutations in GATA4 gene have been implicated in neonatal or childhood-onset diabetes [14]. Human GATA4 is required for endoderm and pancreatic progenitors in a dosage-sensitive manner [15]. Thus, we postulated that regulatory variants of GATA4 gene may alter GATA4 level, conferring susceptibility to T2D development by affecting pancreatic formation and function. In this study, we aimed to investigate whether regulatory variants in GATA4 gene may change GATA4 levels, conferring susceptibility to T2D development. Therefore, the GATA4 gene promoter was genetically and functionally investigated with genomic samples from cohorts of T2D patients and ethnic-matched controls. Regulatory variants of GATA4 gene were identified and functionally analyzed.

## Methods

### Study participants

This was a prospective study. Enrolled T2D patients were newly diagnosed, and the clinical samples were collected from December 2017 to December 2018. The T2D patients (*n* = 255) were recruited from Yanzhou People’s Hospital, Affiliated Hospital of Jining Medical University, Jining Medical University (Jining, Shandong, China). Diagnosis criteria for T2D by WHO (World Health Organization) include fasting blood glucose > 7.0 mmol/L, or 2-h plasma glucose > 11. 0 mmol/L, or HbA1C > 6.5% [16]. T2D patients included 138 males and 117 females. The age range were from 22 to 82 years. Ethnic-matched controls (*n* = 371) were from the subjects receiving routine check-up in the Physical Examination Center in the same hospital, including 197 males and 174 females. The age range were from 21 to 84 years. The controls with heart or kidney diseases were excluded. The patients with Type 1 diabetes (T1D) and the controls with familial history of T1D were excluded from this study. T1D was diagnosed according to the WHO 1999 screening criteria. In this study population, the primary hypertension was diagnosed with recorded systolic pressure > 140 mmHg or diastolic pressure > 90 mmHg or being actively treated for hypertension. The research protocol has been approved by the Ethics Committee of Jining Medical University. This study conforms to the provisions of the Declaration of Helsinki (as revised in Fortaleza, Brazil, October 2013). Informed written consents were obtained from all participants.

### Targeted sequencing

Peripheral leukocytes were isolated and genomic DNAs extracted using QIAamp DNA mini kit (Thermo Fisher Scientific, Waltham, MA, USA). Human GATA4 gene proximal promoter (1028 bp, − 961 bp ~ + 67 bp to the transcription start site) was directly sequenced. Two overlapped DNA fragments covering GATA4 gene proximal promoter, 510 bp (− 961 bp – -451 bp) and 569 bp (− 502 bp – + 67 bp), were generated by PCR. The primers were designed using the human GATA4 genomic sequence (NCBI; NG_008177.2), which were previously reported [17]. PCR products were bi-directionally sequenced. Regulatory variants were identified by aligning the sequences with GATA4 gene promoter.

### Dual-luciferase reporter assay

For functional analysis, wild type and variant GATA4 gene proximal promoters (971 bp, from -932 bp to + 39 bp) were generated by PCR, and inserted upstream to reporter gene-luciferase (pGL3-basic). Human embryonic kidney cells (HEK-293) or rat insulinoma cells (INS-1) were cultured and transiently transfected with expression vectors, together with vector pRL-TK expressing renilla luciferase gene. The transfected cells were grown for 48 h. The cells were then collected and lysed. The dual-luciferases activities of the cell lysates were measured with Promega Glomax 20/20 luminometer. Ratios of luciferase activity over renilla luciferase activity was used to represent transcriptional activity. Wild type GATA4 gene promoter activity was set as 100%. Relative activity of GATA4 gene promoter was obtained. All experiments were repeated three times independently, in triplicate.

### Electrophoretic mobility shift assay (EMSA)

EMSA was performed using LightShift® Chemiluminescent EMSA kit (Thermo Fisher Scientific) according to the procedure. HEK293 and INS-1 cell nuclear extracts were prepared with NE-PER® Nuclear and Cytoplasmic Extraction Reagents (Thermo Fisher Scientific). Protein concentration was determined. Biotinylated probes were double-stranded oligonucleotides (30 bp) with or without regulatory variants. DNA-protein binding was carried out for 20 min (room temperature), and then separated on a 6% polyacrylamide gel. The DNA-protein complexes were subsequently transferred onto a nylon membrane (Thermo Fisher Scientific). Cross-link of oligonucleotides and membrane was conducted and signals detected by chemiluminescence.

### Statistical analysis

SPSS v23.0 was used for statistical analysis in this study. Quantitative data was compared with standard student’s t-test. Frequencies of regulatory variants between two groups were compared with χ^2^ test. *P* < 0.05 was considered as significant.

## Results

### Clinical and biochemical characteristics

Clinical data and biochemical characteristics were shown in Table 1. In this population, percentages of hypertension and smoking in T2D group were significantly higher than those in control group (*P* < 0.05). No significant difference in age, body mass index (BMI), SBP (systolic blood pressure), DBP (diastolic blood pressure), triglyceride (TG), total cholesterol (TC), high density lipoprotein cholesterol (HDL) and low density lipoprotein cholesterol (LDL) existed between two groups (*P* > 0.05).

**Table 1:** Clinical and biochemical characteristics of T2D patients and controls

	Controls(n = 371)	T2D(n = 255)	*P* value
Age (years, mean ± SD)	51.12 ± 12.52	53.00 ± 11.88	0.062
Male (n, %)	197 (53.10%)	138 (54.12%)	0.807
Smoking (n, %)	42 (11.32%)	80 (31.37%)	0.000
BMI (Kg/M^2^)	25.11 ± 3.58	24.86 ± 3.56	0.438
Hypertension (n, %)	94 (25.34%)	97 (38.04%)	0.001
SBP (mmHg)	129.08 ± 21.12	132.28 ± 19.71	0.067
DBP (mmHg)	78.26 ± 13.42	80.19 ± 11.89	0.075
TG (mmol/L)	1.57 ± 1.10	1.66 ± 1.07	0.091
TC (mmol/L)	5.02 ± 0.95	4.88 ± 1.09	0.227
HDL (mmol/L)	1.27 ± 0.30	1.21 ± 0.53	0.359
LDL (mmol/L)	2.91 ± 0.83	2.81 ± 0.86	0.127

*BMI* body mass index, *DBP* diastolic blood pressure, *HDL* high density lipoprotein cholesterol, *LDL* low density lipoprotein cholesterol, *SBP* systolic blood pressure, *TG* triglyceride, *TC* total cholesterol. Quantitative data including age, BMI, TG, TC, HDL and LDL was expressed as mean ± SD

### Identified regulatory variants of GATA4 gene

**Fig. 1:**
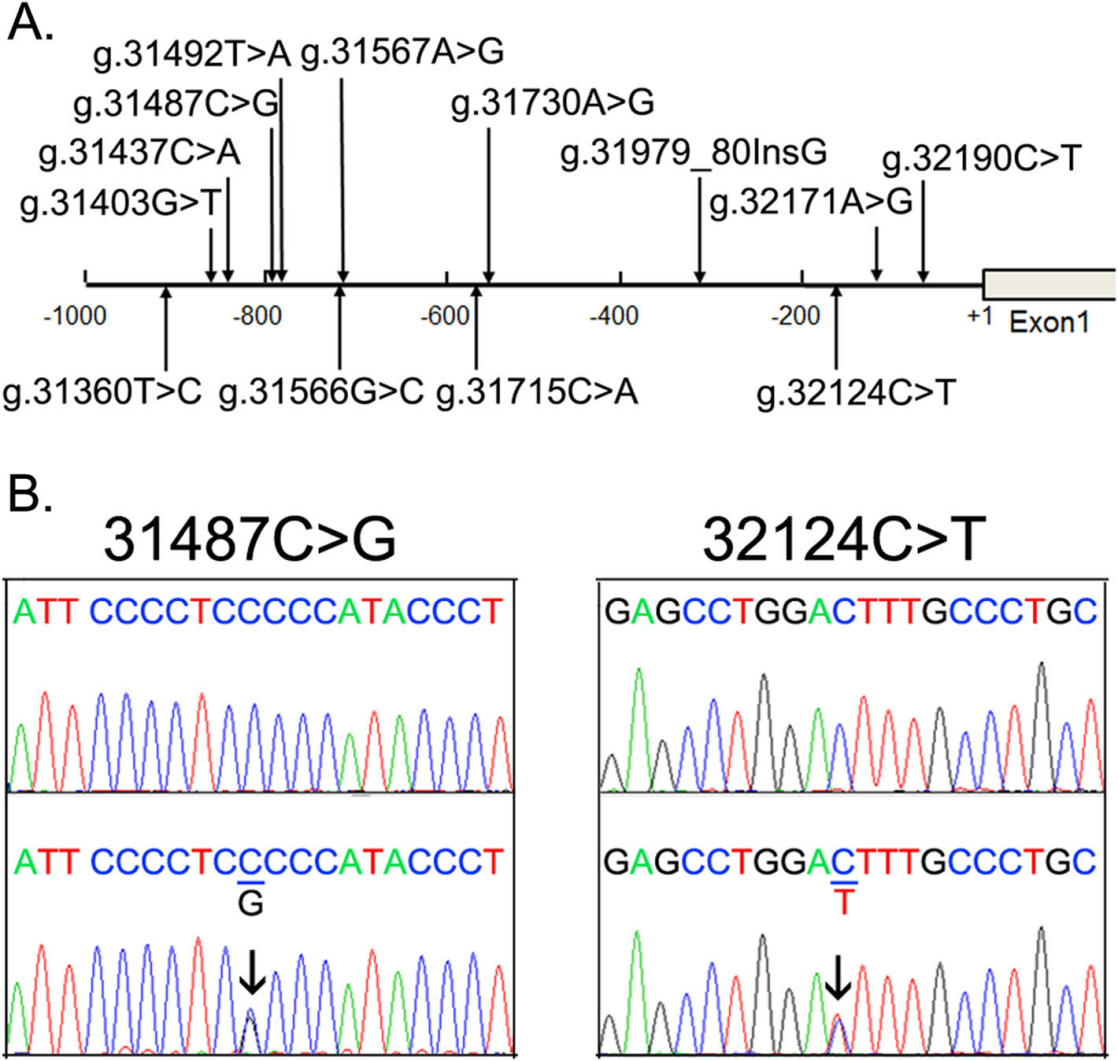
Identified regulatory variants of GATA4 gene. **a**. Locations of the regulatory variants. The transcription start site is at the position of 32,284 (+ 1) in the first exon of the human GATA4 gene (NG_008177.2). **b**. Sequencing chromatograms of the regulatory variants identified in T2D patients. Sequence orientations are all forward. Top panels are wild type and bottom variant sequences. Arrows indicate heterozygous variant

Thirteen regulatory variants, including five SNPs, were identified in GATA4 gene proximal promoter (Fig. 1a and Table 2). One heterozygous variant (g.32124C > T) and one SNP [g.31487C > G (rs1053351749)] were only identified in T2D patients (Fig. 1b). Variant (g.32124C > T) was found in a 52-year old male T2D patient and the SNP [g.31487C > G (rs1053351749)] in a 37-year old female T2D patient. In controls, six heterozygous variants (g.31403G > T, g.31492 T > A, g.31566G > C, g.31567A > G, g.31715C > A and g.32190C > T) and four SNPs [g.31360 T > C (rs372004083), g.31437C > A (rs769262495), g.31730A > G (rs56306152) and g.32171A > G (rs944611351)] were found. In addition, an insertion variant (g.31979_80InsG) was detected in both T2D and controls (*P* > 0.05).

**Table 2:** Regulatory variants in the GATA4 gene promoters in T2D patients and controls

Regulatory variants	Genotypes	Location^a^	Controls (*n* = 371)	T2D (*n* = 255)	*P* value
31,360 T > C (rs372004083)	TC	-924 bp	1	0	–
31403G > T	GT	-881 bp	1	0	–
31437C > A (rs769262495)	CA	-847 bp	1	0	–
31487C > G (rs1053351749)	CG	-797 bp	0	1	–
31,492 T > A	TA	-792 bp	1	0	–
31566G > C	GC	-718 bp	1	0	–
31567A > G	AG	-717 bp	1	0	–
31715C > A	CA	-569 bp	1	0	–
31730A > G (rs56306152)	AG	-554 bp	1	0	–
31979_80InsG	−/G	-304 bp	7	3	0.747
32124C > T	CT	-160 bp	0	1	–
32171A > G (rs944611351)	AG	-113 bp	1	0	–
32190C > T	CT	-84 bp	1	0	–

^a^Variants are located upstream (−) to the transcription start site of GATA4 gene at 32284 of NG_008177.2

### Regulatory variants-affected binding for transcription factors

GATA4 gene promoter was analyzed with JASPAR program (http://jaspar.genereg.net/) to predict regulatory variants-affected putative binding sites for transcription factors. Variant (32124C > T) may abolish binding sites of hepatocyte nuclear factor 4 alpha (HNF4A) and gamma (HNF4G), and create a binding site of Rhox homeobox family member 1. HNF4A has a broad role in glucose homeostasis, and mutations in HNF4A gene has been linked to T2D patients [18]. The SNP [31487C > G (rs1053351749)] may abolish a binding site of SP1 transcription factor, create a binding site of THAP1 (THAP domain containing 1) and modify binding sites of ZNF148 (zinc finger protein 148) and KLF5 (Kruppel like factor 5).

### Functional analysis of regulatory variants

**Fig. 2:**
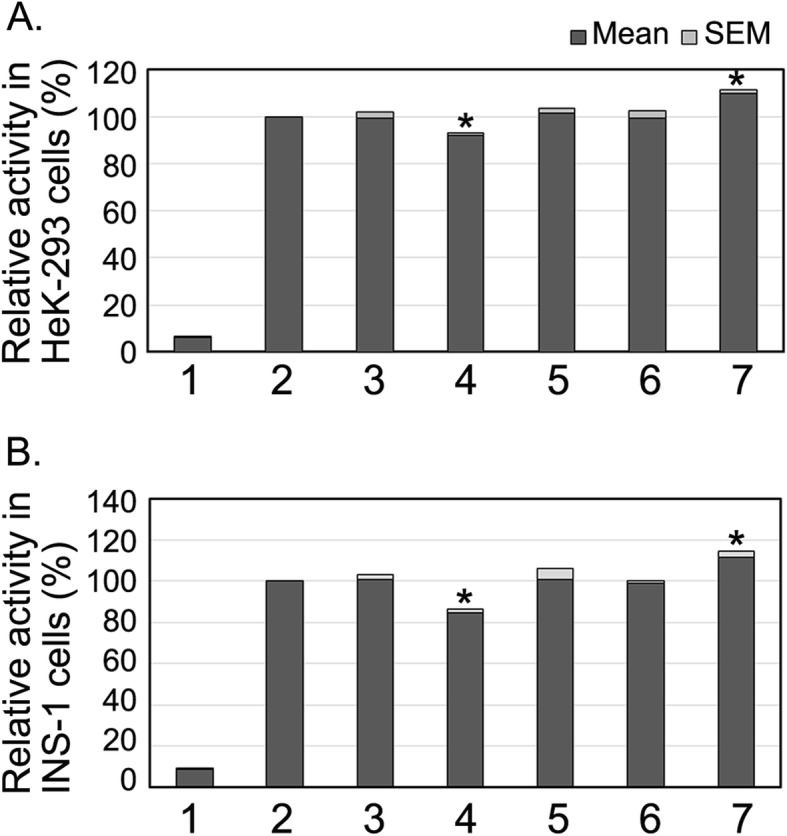
Effects of regulatory variants on GATA4 gene promoter activity. **a**. GATA4 gene promoter activities in HEK-293 cells. **b**. GATA4 gene promoter activities in INS-1 cells. The cultured cells were transfected with expression vector, and dual-luciferase reporter assay was performed. Wild type GATA4 gene promoter activity was set as 100%. The relative activity of variant GATA4 gene promoters was obtained. Lanes 1, pGL3-basic; 2, pGL3-WT; 3, pGL3-31,403 T; 4, pGL3-31487G; 5, pGL3-31566C; 6, pGL3-31567G; 7, pGL3-32,124 T. WT, wild type. *, *P* < 0.01

GATA4 gene regulatory variants were functionally analyzed in cultured cells. The results were shown in Fig. 2. In HEK-293 cells, variant (32124C > T) significantly increased GATA4 gene promoter activity (*P* < 0.01). SNP [31487C > G (rs1053351749)] significantly decreased GATA4 gene promoter activity (P < 0 .01). In contrast, variants (31,403 G > T, 31566G > C and 31567A > G) found in controls did not significantly changed GATA4 gene promoter activity (*P* > 0.05) (Fig. 2a).

To determine tissue-specific effects of the regulatory variants, we examined the GATA4 gene promoter activity in INS-1 cells (Fig. 2b). Variant (32124C > T) significantly increased GATA4 gene promoter activity (*P* < 0.01). SNP [31487C > G (rs1053351749)] significantly decreased GATA4 gene promoter activity (P < 0 .01). As expected, variants (31,403 G > T, 31566G > C and 31567A > G) did not significantly changed GATA4 gene promoter activity (*P* > 0.05). Therefore, these regulatory variants identified in T2D patients affected GATA4 gene promoter activity in both HEK-293 and INS-1 cells, suggesting that their effects was non-tissue specific.

### Regulatory variants-affected transcription factor binding

**Fig. 3:**
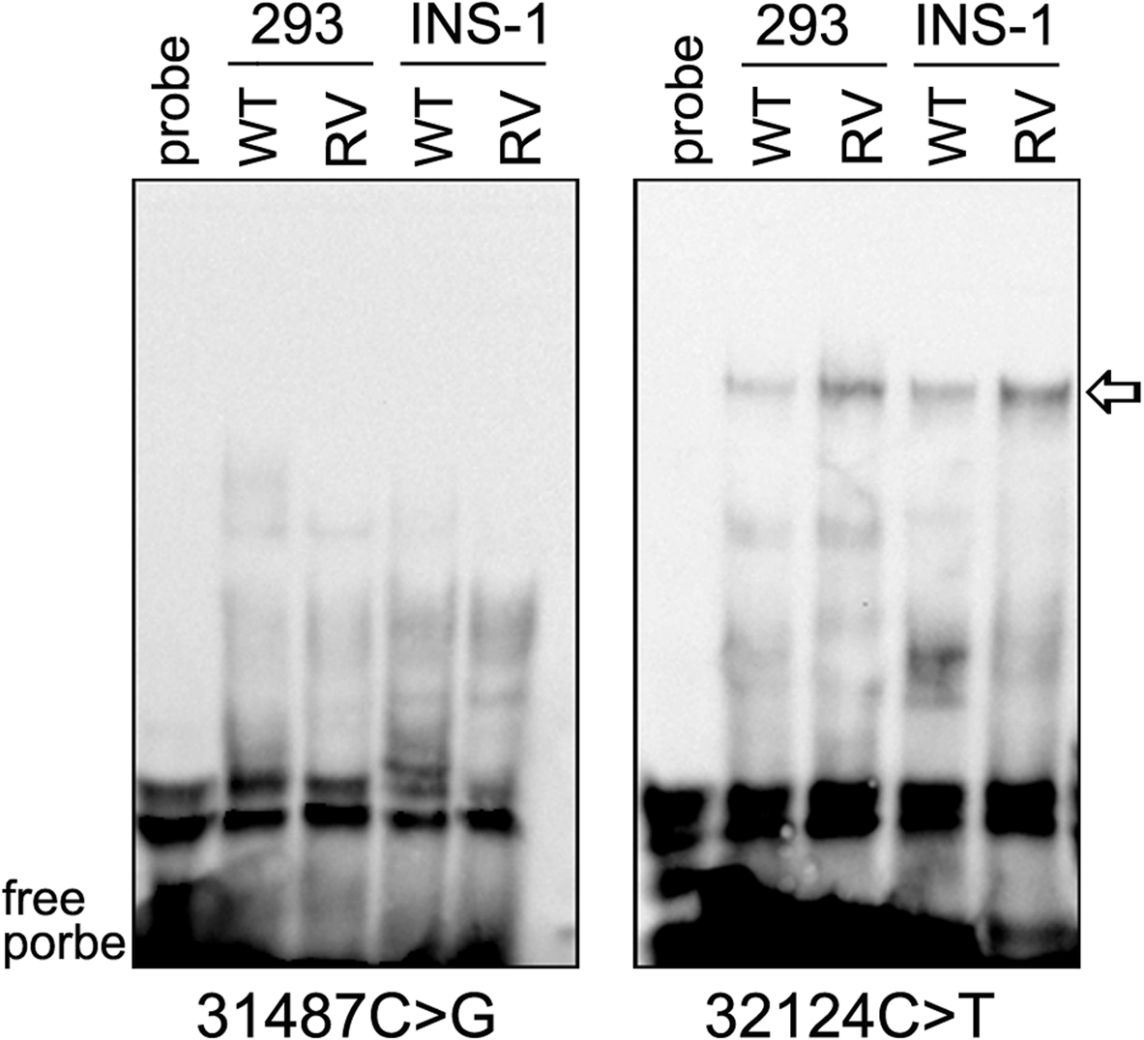
Regulatory variants affect transcription factor binding. EMSA was performed with biotin-labeled oligonucleotide (30 bp) containing regulatory variants and nuclear extracts (HEK-293 and INS-1 cells). Transcription factor binding is indicated with an open arrow. 293, HEK-293. WT, wild type. RV, regulatory variant

EMSA was performed with nuclear extracts of HEK-293 and INS-1 cells. DNA sequences of the oligonecleotides (30 bp) were “GACACATTCCCCTC(C/G)CCCATACCCTGGAAG” for SNP [31487C > G (rs1053351749)], and “CCCCAGAGCCTGGA(C/T)TTTGCCTGCTGGGGG” for variant (32124C > T). The results showed that in both line cells, the variant (32124C > T) evidently enhanced the binding ability of a transcription factor (Fig. 3). Since variant (32124C > T) increased GATA4 gene promoter activity, this transcription factor probably functions as an activator. The effect of SNP [31487C > G (rs1053351749)] on transcription factor binding was not detected, likely due to low level of the transcription factors or EMSA sensitivity limit.

## Discussion

Manipulation of GATA4 gene expression by targeting its promoter with genetic or therapeutic approaches might provide a potential way for clinical purposes. GATA4 gene mutations have been associated with several human diseases, including congenital heart disease, coronary artery disease, hypertension, type 1 diabetes and various cancers [14, 19]. A GATA4 mutation has been found in a child with atrial septal defect and neonatal diabetes caused by pancreatic agenesis [20]. In human ectopic pancreatic tissues, GATA4 gene expression is downregulated [11]. In previous studies, we have found several GATA4 gene regulatory variants in patients with congenital heart disease [17]. In this study, we identified two functional regulatory variants of GATA4 gene in T2D patients. Considering the complex genetic heterogeneity in T2D etiology, GATA4 gene regulatory variants may probably contribute to the T2D development as a rare risk factor.

The human GATA4 gene is mapped to chromosome 8p23.1-p22, and contains seven exons [21]. In normal human tissues, GATA4 is highly detected in heart, liver, pancreas, stomach, small intestines, ovaries and testes [6]. There are conserved GC-box, E-box, AP-1 and GATA motif in the GATA4 gene promoter region [22]. FOXA2 regulates GATA4 gene expression through an intronic enhancer [23]. A distant enhancer of GATA4 gene is bound by pancreatic duodenal homeobox 1, directing its expression in the mouse endoderm [24]. In this study, conserved binding motifs for transcription factors in GATA4 gene promoter were not disrupted by identified regulatory variants. Therefore, the identified regulatory variants may change GATA4 gene expression levels by altering the binding of transcription factors.

Recent studies on genomic occupancy of GATA4 indicates that GATA4 exhibits cell-type-specific binding [25]. To date, few GATA4-interacting proteins and downstream targets have been identified in the pancreatic formation. GATA4 and GATA6 coordinate to regulate embryonic pancreatic regulators [9, 10]. GATA4 and GATA6 inhibit hedgehog signaling to regulate pancreatic endoderm specification [26]. GATA4 co-ocupyies genomic regions with TCF7L2, and represses TCF7L2/β-catenin complex in adult heart [27]. Interestingly, TCF7L2 gene polymorphisms are linked to type 1 diabetes and T2D [28]. In addition, GATA4 contributes to the regulation of pancreas development by initiating pancreatic gene regulation, and is involved in regulating pancreatic glucagon gene expression [29]. In mouse neuroendocrine tumor derived cells, GATA4 increases insulinotropic polypeptide gene expression [30]. Collectively, GATA4 plays essential roles for beta cell activity and pancreatic development. As GATA4 is a dosage-sensitive regulator, deficient or excessive GATA4 may disrupt pancreatic β-cell function.

In conclusion, two functional regulatory variants in GATA4 gene were identified in T2D patients. Our study suggested that GATA4 gene regulatory variants may probably contribute to the T2D development as a rare risk factor by influencing beta cell development and activity.

Since GATA4 is involved in the pancreatic development and acts in a dose-sensitive manner, we first investigated the regulatory variants of GATA4 gene in T2D patients, which was the strength of the study. As this was a primary study, the sample size was relatively small, which was the limitation of the study. Further larger studies are needed to confirm the finding from this study.

## Data Availability

The datasets used and/or analyzed during the current study are available from the corresponding author on reasonable request. The novel variants of GATA4 gene promoter has been deposited in the NCBI SNP database. The NCBI Sub_SNP numbers are following, 31403G > T (ss2137544185), 31492 T > A (ss5236862208), 31566G > C (ss5236862209), 31567A > G (ss5236862210), 31715C > A (ss5236862211), 32124C > T (ss5236862213), 32190C > T (ss5236862214) and 31979_80InsG (ss5236862212).

## Abbreviations

GATA4: Transcription factor GATA4
GATA6: Transcription factor GATA6
HEK-293: Human embryonic kidney cells
HNF4A: Hepatocyte nuclear factor 4 alpha
HNF4G: Hepatocyte nuclear factor 4 gamma
INS-1: Rat insulinoma cells
KLF5: Kruppel like factor 5
THAP1: THAP domain containing 1
T2D: Type 2 diabetes
ZNF148: Zinc finger protein 148
